# Chameleon-Inspired Colorimetric Sensors for Real-Time Detections with Humidity

**DOI:** 10.3390/mi14122254

**Published:** 2023-12-18

**Authors:** Yu-Hsuan Cheng, Ching-Te Kuo, Bo-Yao Lian

**Affiliations:** 1 Department of Mechanical and Electro-Mechanical Engineering, National Sun Yat-sen University, Kaohsiung 80424, Taiwan; as557666@gmail.com; 2 Department of Biological Sciences, National Sun Yat-sen University, Kaohsiung 80424, Taiwan; a0966158120@gmail.com

**Keywords:** vapor sensor, colorimetric, humidity, ethanol

## Abstract

In recent decades, vapor sensors have gained substantial attention for their crucial roles in environmental monitoring and pharmaceutical applications. Herein, we introduce a chameleon-inspired colorimetric (CIC) sensor, detailing its design, fabrication, and versatile applications. The sensor seamlessly combines a PEDOT:PSS vapor sensor with a colorimetric display, using thermochromic liquid crystal (TLC). We further explore the electrical characteristics of the CIC sensor when doped with ethylene glycol (EG) and polyvinyl alcohol (PVA). Comparative analyses of resistance change rates for different weight ratios of EG and PVA provide insights into fine-tuning the sensor’s responsiveness to varying humidity levels. The CIC sensor’s proficiency in measuring ambient humidity is investigated under a voltage input as small as 2.6 V, capturing resistance change rates and colorimetric shifts at relative humidity (RH) levels ranging from 20% to 90%. Notably, the sensor exhibits distinct resistance sensitivities of 9.7 mΩ (0.02% ∆R/R_0_)/%RH, 0.5 Ω (0.86% ∆R/R_0_)/%RH, and 5.7 Ω (9.68% ∆R/R_0_)/%RH at RH 20% to 30%, RH 30% to 80%, and RH 80% to 90%, respectively. Additionally, a linear temperature change is observed with a sensitivity of −0.04 °C/%RH. The sensor also demonstrates a colorimetric temperature sensitivity of −82,036 K/%RH at RH 20% to 30% and −514 K/%RH at RH 30% to 90%, per captured image. Furthermore, real-time measurements of ethanol vapor with varying concentrations showcase the sensor’s applicability in gas sensing applications. Overall, we present a comprehensive exploration of the CIC sensor, emphasizing its design flexibility, electrical characteristics, and diverse sensing capabilities. The sensor’s potential applications extend to real-time environmental monitoring, highlighting its promising role in various gas sensing fields.

## 1. Introduction

In recent years, scientific research has increasingly centered on advancing sensor technology, driven by its extensive applications in environmental monitoring, healthcare, industrial processes, and space environments [1,2,3,4,5,6]. Various sensing materials and mechanisms have been investigated to augment the capabilities of humidity sensors, encompassing resistance-based [7], capacitance-based [8,9], refractive index-based [10,11], bulk acoustic wave resonator-based [12], and field-effect transistor-based sensors [13]. The prominence of resistive and capacitive sensors among these types is attributed to their affordability, ease of fabrication, biomimetic phage nanostructures [14,15], environmental friendliness, and seamless integration with electronic circuits [16,17,18,19,20].

Among the frequently studied sensing materials, poly(3,4-ethylenedioxythiophene) (PEDOT) has emerged as a highly conductive and transparent polymer, capable of dispersing in water when combined with poly(styrenesulfonate) (PSS) [21,22,23]. PEDOT:PSS has demonstrated applications in various organic electronic devices, including solar cells, transistors, and thermoelectric devices, positioning it as a promising candidate for humidity sensors [24,25,26,27,28,29,30]. For instance, the integration of graphene oxide (GO) and PEDOT:PSS in the development of small flexible relative humidity (RH) sensors has exhibited high sensitivity of 1.22 nF/%RH under 1 kHz impedance recording [20]. Furthermore, the combination of inter-digital transducer (IDT) with GO and C_15_H_15_N_3_O_2_ (Methyl Red) has expanded the detection range from 0% RH to 100% RH [31]. In the realm of humidity sensors, the utilization of quartz crystal microbalance (QCM) stands out, particularly emphasizing the role of PEDOT:PSS mixed with polyvinyl alcohol (PVA) as a hydrophilic polymer for modifying QCM sensors. This integration has contributed to an exceptional sensitivity of up to 33.56 Hz/%RH [17]. Nevertheless, conventional humidity sensors often employ expensive instruments like LCR meters to read out capacitance or impedance signals, which may pose challenges for user-friendly and point-of-care applications due to their inherent high power requirements.

To address these drawbacks, we present a chameleon-inspired colorimetric (CIC) sensor that seamlessly integrates a PEDOT:PSS vapor sensor with a colorimetric display, using thermochromic liquid crystal (TLC). This study delves into the electrical characteristics of the CIC sensor when doped with ethylene glycol (EG) and polyvinyl alcohol (PVA). Comparative analyses of resistance change rates for different weight ratios of EG and PVA offer insights into fine-tuning the sensor’s responsiveness to varying humidity levels. The ambient humidity can be easily evaluated based on the correspondence of TLC colorimetry captured by a camera. Additionally, real-time measurements of ethanol vapor with varying concentrations demonstrate the sensor’s versatility in gas sensing applications. Note that the requirement of voltage input could be set as small as 2.6 V (relevant to 0.5 μW).

Essentially, our CIC sensor overcomes the constraints of traditional humidity sensors by providing a user-friendly, cost-effective, and adaptable solution. The incorporation of a colorimetric display not only elevates the sensing experience but also aligns with the broader objective of advancing environmental monitoring technologies. This innovation will enable individuals to promptly assess their ambient humidity by interpreting the color mapping on the TLC display. Furthermore, it will open avenues for evaluating the concentration of ethanol vapors in the surroundings, facilitating in situ drunk-driving tests. Our work not only builds on the humidity-mediated resistance property of the CIC sensor but also explores the influence of different materials and fabrication techniques, contributing to the broader landscape of humidity sensing research.

## 2. Experimental Section

### 2.1. Synthesis of CIC Sensor

**Figure 1:**
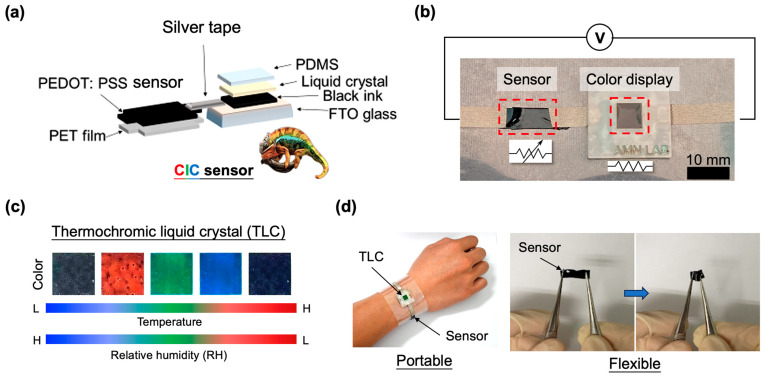
Design of the chameleon-inspired colorimetric (CIC) sensor. (**a**) Schematic depicting the fabrication process of the CIC sensor. (**b**) The end product of the CIC sensor, featuring an integrated PEDOT:PSS vapor sensor and a colorimetric display composed of thermochromic liquid crystal (TLC). Scale bar: 10 mm. (**c**) Representative images illustrating the colorimetric variations corresponding to changes in ambient temperature and humidity. (**d**) Images showcasing the wearability, portability, and flexibility of the CIC sensor.

Figure 1a depicts the design concept and synthesis process of the proposed chameleon-inspired colorimetric (CIC) sensor, comprising a humidity sensing unit and a thermochromic liquid crystal (TLC) display. The humidity sensing unit was created by printing PEDOT:PSS (1.25 wt%; thickness = 36 µm; Clevios TM PH 1000, Heraeus, Hanau, Germany) with and without doping solutions onto a customized PET film (20 mm by 10 mm). Doping materials, such as EG (0 to 35 wt%; EG; Xilong Scientific Co., Ltd., Shantou, China) and PVA (0 to 40 wt%), were thoroughly mixed with the primary PEDOT:PSS solution before printing. After heating at 60 °C for 3 h, the sensing block was formed. For TLC display synthesis, the sprayable liquid crystal ink (SFXC, NEC, Newhaven BN99BA, UK) was screen-printed on a fluorine-doped tin oxide (FTO) glass (SGAST0002-0702PK, STAREK Scientific Co., Ltd., Taipei, Taiwan) pre-coated with black ink (acrylic inks, Culture Hustle, London, UK). The TLC film was allowed to dry completely for an additional 3 h at 60 °C, maintaining a thickness of approximately 50 µm. Subsequently, a thin film of polydimethylsiloxane (PDMS) prepolymer covered the TLC surface, protecting it from ambient humidity and particle contamination. In the final step, the two blocks, the sensor film, and the colorimetric display were connected in series, using conductive silver (Ag) adhesives, and the entire assembly was affixed to a customized PET film. The resulting CIC sensor is illustrated in Figure 1b.

### 2.2. Experimental Setup of Vapor Control Chamber

**Figure 2:**
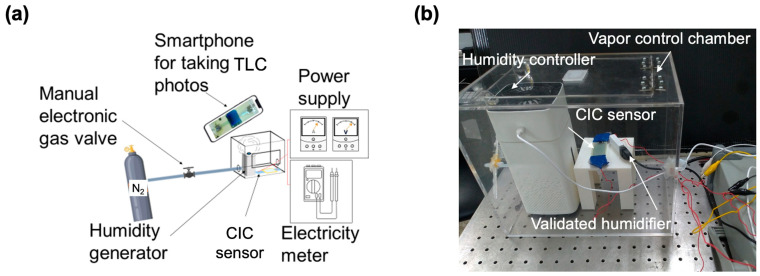
Experimental setup for CIC sensor evaluation. (**a**) Schematic illustrating the observation chamber designed for real-time measurements of ambient humidity. (**b**) Photograph providing an overview of the complete experimental setup. A commercial humidifier is employed to validate the readout obtained from the CIC sensor.

The experimental arrangement for assessing ambient humidity utilizing the CIC sensor is illustrated in Figure 2a. This setup incorporated an airtight box designed to facilitate the inflow of tested vapors. The system employed a humidifier (p01_05242554, AHOYE, New Taipei City, Taiwan) and a gas control unit (nitrogen gas and digital gas valve (TF-6050D, TOKYO KEISO TAIWAN Co., Ltd., Hsinchu, Taiwan)) to generate varied humidity levels ranging from 20% RH to 90% RH. For ethanol vapor testing, the humidifier was substituted with an ethanol vapor generator. Additionally, a commercial humidity sensor (TEMPerHUM, ANJUN, Taoyuan, Taiwan) served for validation in comparison with the CIC sensor. The resistance change in the CIC sensor and the temperature variation in the TLC display were recorded using a commercial multimeter (GDM-8342, Good Will Instrument Co., Ltd., New Taipei City, Taiwan) integrated with a thermal coupler. All the investigations were performed with 2 or 3 individual CIC sensors. Figure 2b provides an overview of the entire setup of the vapor control chamber.

### 2.3. Image Recording for Chromatic Analysis Using CIC Sensor

To showcase the potential for portable image capture, we utilized a commercial iPhone 12 (with 12 megapixels), ensuring uniform conditions in terms of exposure time and focus length. While auto-exposure and auto-flash features were deactivated, a fixed exposure time of 1/30 s was maintained. Following this, the recorded images were subjected to analysis using the ImageJ software (ImageJ 1.54f), a robust and freely available image processing tool. Initially, the images underwent auto brightness/contrast processing to enhance the chromaticity levels of red (R), green (G), and blue (B). Next, the assessed RGB levels were converted to obtain values in the CIE 1931 color space (Y_xy_) and color temperature (unit: K). Finally, the obtained values were plotted on the CIE 1931 chromaticity diagram [32], and the chromaticity level was expressed as a percentage, using the following formula: chromaticity level (%) = R, G, or B levels/255.

## 3. Results and Discussion

### 3.1. Characteristics of TLC Display

The fabricated TLC display exhibits a highly responsive chromatic shift corresponding to changes in ambient humidity and temperature, as illustrated in Figure 1c. Moreover, the proposed CIC sensor showcases notable portability and flexibility, as depicted in Figure 1d. Bending the sensor at a 180° angle led to a notable change in its inherent resistance, shifting from an average of 47 Ω to 69 Ω during pre-conditional testing at RH 48%. Similarly, with an increase in humidity to RH 68%, the resistance exhibited a change from 56 Ω to 75 Ω. Consequently, the calculated resistance change rates under the bending effect were 1.47 at RH 48% and 1.34 at RH 68%. Importantly, the change rates under 0° and 180° bending effects were 1.19 and 1.09, respectively, calculated from RH 68% to 48%. This suggests that the sensor’s responsiveness to bending events may not yield a significantly varied outcome.

**Figure 3:**
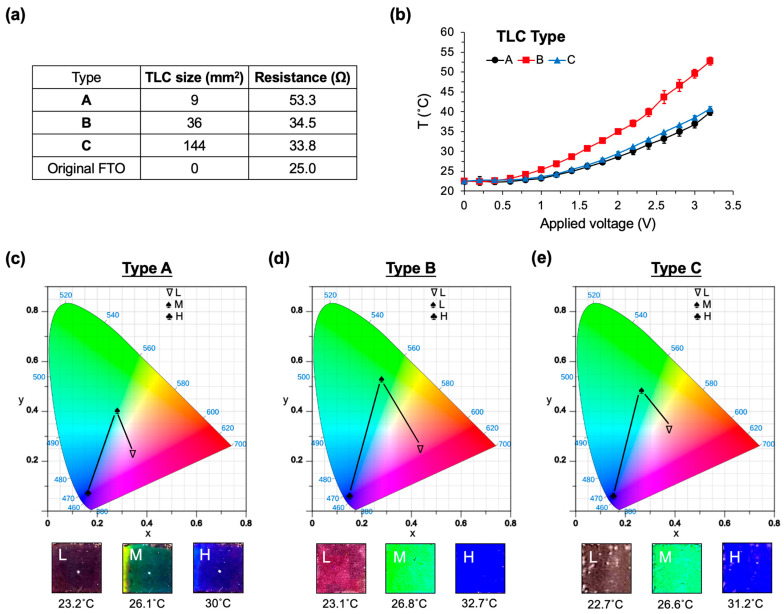
Characteristics of the TLC display in the CIC sensor. (**a**) A comparison of display sizes and corresponding resistances for the TLCs (Types A, B, and C). The main substrate utilizes fluorine-doped tin oxide (FTO) with a native resistance of 25 Ω. (**b**) Comparison of applied voltages and the resulting temperature changes for the three TLC displays. (**c**) CIE 1931 chromaticity diagram of Type A TLC, along with actual images at 23.2 (denoted as L), 26.1 (M), and 30 (H) °C. (**d**) Type B TLC, featuring actual images recorded at 23.1 (L), 26.8 (M), and 32.7 (H) °C. (**e**) Type C TLC, with actual images recorded at 22.7 (L), 26.6 (M), and 31.2 (H) °C. Voltages are applied in the range of 0 to 3.2 V.

To explore the colorimetric characteristics of the TLC display, we introduce three distinct display sizes (Types A, B, and C) in Figure 3a. These display types feature areas of 9, 36, and 144 mm^2^, with measured resistances of 53.3, 34.5, and 33.8 Ω, respectively, compared to the native resistance of the original FTO substrate, which is 25 Ω. Figure 3b presents a comparative analysis of the temperature profiles among the three TLC types across a voltage input range of 0 to 3.2 V. Notably, Type B TLC exhibits a relatively sensitive response to power input, displaying a discernible temperature gradient compared to other designs.

When examining the colorimetric changes using CIE 1931 chromaticity [32], Type B TLC contributes to a broader chromatic range, spanning from low temperature (L; 23.1 °C), through middle temperature (M; 26.8 °C), to high temperature (H; 32.7 °C), as depicted in Figure 3c–e. Intriguingly, the chromatic levels of the TLC manifest as nearly red at low temperatures (22.7~23.2 °C), green at middle temperatures (26.1~26.8 °C), and blue at high temperatures (30~32.7 °C).

The color display’s recognition plays a pivotal role in image-based humidity classification, whether through a camera or visual observation. Opting for a smaller TLC display enhances the user experience of portable devices, contributing to reduced weight and size. In summary, the Type B TLC display, sized at 36 mm^2^, exhibits exceptional responsiveness in terms of temperature changes relative to power input or the broader recognition of visible colors. As a result, we chose the Type B display for in-depth exploration in both humidity sensing and ethanol vapor detection.

### 3.2. Characteristics of PEDOT:PSS Humidity Sensor with and without Doping Materials

**Figure 4:**
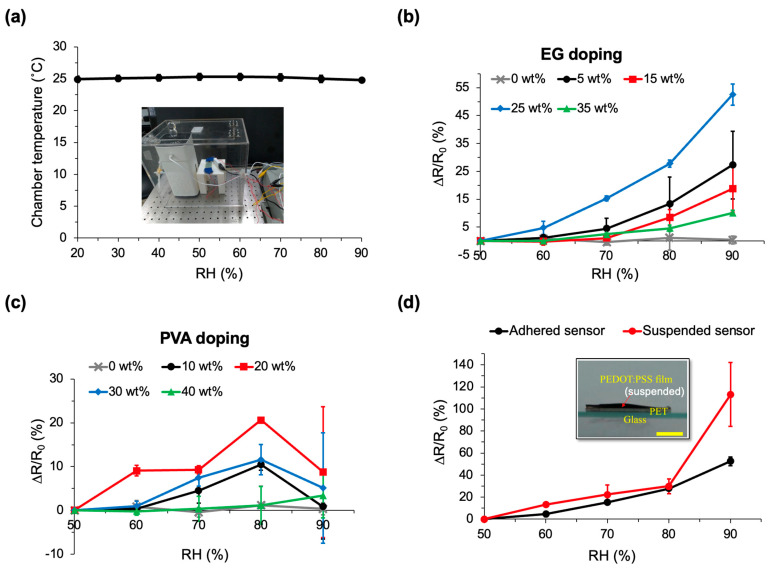
Electrical characteristics of the CIC sensor with EG and PVA Doping. (**a**) Recording the temperature distribution of the vapor control chamber across relative humidity (RH) levels from 20% to 90%. (**b**) Comparative analysis of resistance change rates obtained from PEDOT:PSS vapor sensors doped with varying weight ratios of ethylene glycol (EG) (ranging from 0 to 35 wt%) at RH 50% to RH 90%. (**c**) Comparative assessment of resistance change rates derived from PEDOT:PSS vapor sensors doped with different weight ratios of polyvinyl alcohol (PVA) (ranging from 0 to 40 wt%) at RH 50% to RH 90%. (**d**) Electrical characteristics comparison between adhered and suspended PEDOT:PSS films doped with 25 wt% EG. The inset image provides details of the suspended vapor sensor film. The voltage input is 2.6 V. Scale bar: 5 mm.

To assess the humidity sensing characteristics of the PEDOT:PSS-mediated sensor, a vapor testing chamber is established, maintaining a stabilized chamber temperature within the range of 20% to 90% RH (Figure 4a). Two dopants, EG and PVA, are employed for this verification, yielding noteworthy results. When doped with 25 wt% EG, the sensor exhibits a remarkably high sensitivity in measuring the resistance change rate (∆R/R_0_) relative to chamber humidity, especially compared to other EG dopings (Figure 4b). This rate is measured using a multimeter. The resistance change rate varied from 0% at RH 50% to 52.5% at RH 90%. In contrast, the original PEDOT:PSS humidity sensor demonstrates a minimal response, with a mere 1.2% ∆R/R_0_ at RH 80%, and no significant difference across other humidity levels. Conversely, PVA doping does not enhance detection sensitivity to humidity across all PVA doping groups (ranging from 10 to 40 wt%), as illustrated in Figure 4c.

As a result, the composite sensor, specifically PEDOT:PSS/25 wt% EG, was chosen for subsequent testing with ambient humidity. Figure 4d further illustrates that the pseudo-3D (suspended) sensor outperformed the adhered sensor in humidity sensing, particularly beyond RH 80%, showing an evaluated ratio of 113% compared to 52.5% from the original/adhered sensor type. However, within the RH range of 50% to 80%, no significant difference is observed between the two sensor types. Consequently, the suspended PEDOT:PSS/25 wt% EG sensor was selected for further investigation across broader ranges of ambient humidity.

### 3.3. Real-Time Detection with Humidity Using CIC Sensor

**Figure 5:**
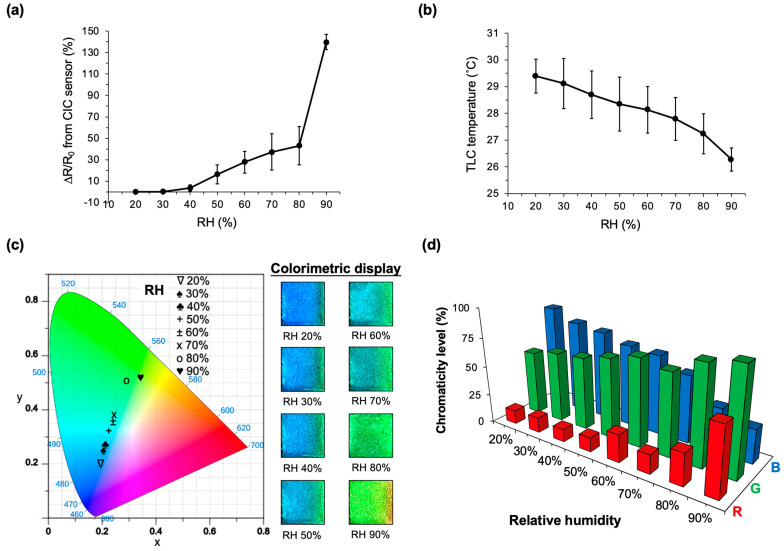
Measuring ambient humidity using the CIC sensor. (**a**) Recording the resistance change rates of the sensor at RH levels ranging from 20% to 90%. (**b**) Displaying the corresponding surface temperature changes in the TLC throughout all humidity testing. (**c**) CIE 1931 chromaticity diagram of the CIC sensor, accompanied by actual images captured at RH levels from 20% to 90%. (**d**) Comparative analysis of the chromaticity levels (R, G, and B) derived from images in (**c**) at RH levels from 20% to 90%. The testing sensor is composed of PEDOT:PSS doped with 25 wt% EG. The voltage input is 2.6 V.

To explore the sensing capabilities across broader ranges of ambient humidity using the integrated CIC sensor (as depicted in Figure 1b), tests were conducted inside the vapor control chamber (illustrated in Figure 4a). It employs a constant voltage input of 2.6 V, resulting in an overall input power of approximately 0.5 μW. The PEDOT:PSS film, known for absorbing ambient vapor and modulating internal electrical transduction [33], can be modeled as an adjustable resistor relative to humidity. Figure 5a,b illustrate the resistance change rate (∆R/R_0_) and TLC display temperature measured from the integrated CIC sensor, respectively, across testing humidity levels ranging from 20% RH to 90% RH.

The sensor exhibits distinct resistance sensitivities of 0.02% ∆R/R_0_ (9.7 mΩ)/%RH, 0.86% ∆R/R_0_ (0.5 Ω)/%RH, and 9.68% ∆R/R_0_ (5.7 Ω)/%RH at RH 20% to 30%, RH 30% to 80%, and RH 80% to 90%, respectively (Figure 5a). Additionally, a linear temperature change is observed with a sensitivity of −0.04 °C/%RH (Figure 5b). Colorimetric recognition using the CIC sensor and CIE 1931 chromaticity among the testing humidity levels (RH 20% to RH 90%) was investigated. Figure 5c shows that the colorimetric images that were recorded from the tested smartphone and analyzed using ImageJ exhibit comparable and recognizable RGB colors, either from the transformed CIE 1931 Y_xy_ levels or directly from human eyes.

The sensor also demonstrates a colorimetric temperature sensitivity of −82,036 K/%RH at RH 20% to 30% and −514 K/%RH at RH 30% to 90% per captured image. Figure 5d compares the chromaticity levels (i.e., the relevant ratios of R, G, and B) analyzed from the TLC display of the sensor among the humidity ranges of RH 20% to RH 90%. Notably, a prominent expression with a red color is observed at RH 90%, corresponding to the largest resistance change and the lowest temperature level measured in Figure 5a,b. This suggests that the vapor sensor, at RH 90%, absorbs abundant water vapors, decreasing its electrical conductivity and attenuating the Joule heating on the TLC display. In contrast, within the range of RH 20% to RH 50%, the blue color dominates the chromatic expression of the TLC, indicating that Joule heating primarily controls this expression at a higher temperature level resulting from a lower resistance change.

To assess the response time of the CIC sensor, considering both resistance and chromaticity changes, measurements were conducted under pre-conditional humidity conditions ranging from RH 48% to RH 68%. The time required for a stable assessment of resistance change was determined to be 15.3 ± 2.5 s, while the corresponding time for the TLC chromaticity change was measured at 54.8 ± 6.1 s. This indicates a lag time of 39.5 s between evaluating resistance changes and color changes. The CIC sensor, predominantly covered with a thin waterproof PDMS layer, except for the PEDOT:PSS/EG sensor area, demonstrated robust and stable functionality even under high-humidity conditions at 90%, with a calculated coefficient of variation (CV) of 5% for the measured resistance change.

Overall, these demonstrations strongly confirm that the proposed CIC sensor has the potential for the real-time detection of ambient humidity, which is evident from both electrical changes and color recognition.

### 3.4. Real-Time Detection with Ethanol Vapor Using CIC Sensor

**Figure 6:**
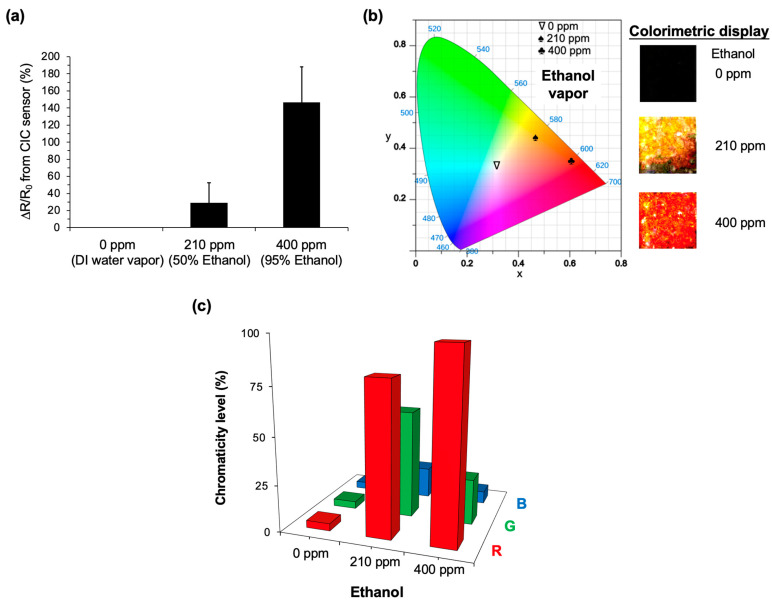
Real-time measurement of ethanol vapor with different concentrations by the CIC sensor. (**a**) Comparative analysis of the electric resistance measured at ethanol concentrations of 0 ppm (controlled by DI water vapor), 210 ppm (by 50% ethanol), and 400 ppm (by 95% ethanol). (**b**) CIE 1931 chromaticity diagram of the CIC sensor, accompanied by actual images captured at ethanol levels ranging from 0 to 400 ppm. (**c**) Comparative analysis of the chromaticity levels (R, G, and B) derived from images in (**b**). The testing sensor is composed of PEDOT:PSS doped with 25 wt% EG. The voltage input is 2.6 V.

For the proof-of-conceptual demonstration of ethanol vapor sensing using the CIC sensor, we followed the methods described earlier. The results are intriguing. Figure 6a compares the resistance change rate of the sensor under different ethanol concentrations—0 ppm (controlled by DI water vapor), 210 ppm (occupied by a 50% ethanol solution), and 400 ppm (by 95% ethanol). The change rate of resistance is determined to be up to 146.6% at 400 ppm ethanol and 29.6% at 210 ppm ethanol, compared to the control vapor. Figure 6b further compares the colorimetric images and the relevant CIE 1931 chromaticity based on the results from Figure 6a. The control vapor (0 ppm ethanol) exhibits a nearly black color, as confirmed by analyzing its chromaticity level shown in Figure 6c. Importantly, a relatively high level of red color is present in the 400 ppm ethanol, whereas a nearly yellow-expressed image is observed in the 210 ppm ethanol. This suggests that the absorption of ethanol vapor within the sensor could result in an increase in electrical resistance, attenuating the Joule heating to the TLC display and thus acquiring a red-color-dominated image. Note that a constant voltage input of 2.6 V is employed for the sensor.

A previous study revealed that the addition of PVA to PEDOT:PSS could enhance the wide-range relative humidity sensor, showing a maximum change in AC impedance from 137 kΩ to 110 kΩ and in capacitance from 96 pF to 124 pF from RH 0% to RH 80% [16]. Another study demonstrated that blending EG and Triton X-100 with PEDOT:PSS could significantly decrease the sheet resistance of the polymer composite from 1.5 kΩ/sq down to 0.02 kΩ/sq [34]. In comparison to our results shown in Figure 4c, the PVA dopant does not efficiently enhance the sensing against humidity. It is suggested that our measurement of electric resistance is in DC, whereas the previous study uses AC to record the impedance. As PVA is water-affinitive [35], the PEDOT:PSS/PVA composite could absorb abundant water vapors at a high humidity condition compared to native PEDOT:PSS. This results in an increased capacitance and thus acquires a decreased impedance, measured from an AC impedance analyzer. For DC resistance measurement, the abundant water vapor content could attenuate the electric conductivity within the composite, thus resulting in the unexpected results shown in Figure 4c. Remarkably, the PEDOT:PSS/25 wt% EG composite strongly enhances the detection sensitivity to humidity, as shown in Figure 4b. It suggests that blending with EG could increase the electrical conductivity and thus improve the detection sensitivity used in our sensor. Note that our sensor does not require expensive instruments for detection; instead, it records the resistance change or recognizes the colorimetry by eye with a power supply as low as 0.5 µW, covering relative humidity from RH 20% to 90%.

**Figure 7:**
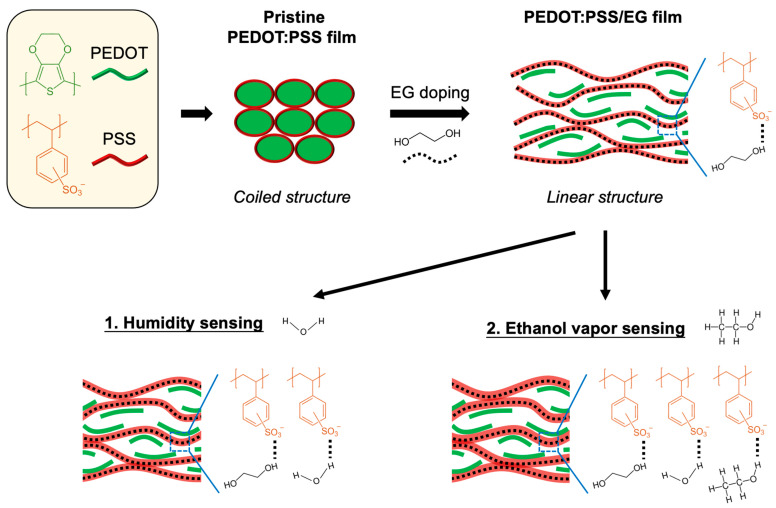
Schematic illustration of PEDOT:PSS with secondary EG doping for sensing both humidity and ethanol vapor.

The mechanism governing distinct resistance and colorimetric changes in response to humidity and ethanol vapors for the CIC sensor is depicted in Figure 7. In the pristine PEDOT:PSS, water-insoluble PEDOT, with positive charges, adheres to segments of PSS anion chains, possessing negative charges, through Coulombic attraction, forming a coiled structure. The hydrophilic PSS-enriched shell shields the hydrophobic PEDOT-enriched core from water through colloidal dispersion [36]. Doping PEDOT:PSS with EG induces a conformational change in the polymer backbone chain from coiled (benzoid) to linear structures (quinoid), as evidenced by the Fourier-transformation infrared spectroscopy (FTIR) analysis, which shows a C=C stretching of the quinoid structure that shifted from 1620 to 1632 cm^−1^ [37]. This conformational change is implied to enhance the conductivity of the composite sensor. For humidity sensing, the diffusion of water vapor (H_2_O) into the sensor may lead to film swelling due to the absorption between PSS/EG and H_2_O through hydrogen bonding, resulting in a mixture of H_3_O^+^ and PSS(SO_3_)^−^. Film swelling could attenuate charge carrier mobility due to the increased distance between PEDOT and PSS chains [38]. This aligns with our observations, indicating that higher humidity increases the resistance change in the CIC sensor (Figure 4) and decreases Joule heating on the TLC display, resulting in a red-shifted expression (Figure 5). Similarly, for ethanol vapor sensing, the abundance of hydroxyl groups in ethanol makes it susceptible to hydrogen bonding with PSS/EG. This leads to additional film swelling compared to water vapor alone, suggesting that higher resistance changes and, consequently, red-shifted color mapping can be measured, as shown in Figure 6.

To comprehensively compare the performance of our CIC sensor with previously reported humidity sensors, we compiled information in Table 1. In general, most studies utilize external instruments to collect sensing data, such as an LCR meter [20], QCM [39], impedance analyzer [40], and digital multimeter [18]. These instruments are typically expensive and not suitable for portable device applications. Our developed CIC sensor addresses this issue by utilizing a camera or a smartphone for image capture and automatic analysis. Furthermore, the relative humidity can be directly recognized through visual inspection. As a result, the CIC sensor not only provides practical detection sensitivity to humidity but also can be employed for the real-time detection of ethanol vapors.

The current limitations of our CIC sensors encompass compatibility with different smartphones or cameras and sensitivity to varying environmental temperatures. Higher ambient temperatures, for instance, could potentially modify the initial color expression on the TLC display, introducing variations in standard color recognition established in this study. In future applications, we intend to implement an image processing algorithm that is capable of standardizing images captured under diverse temperature conditions, ensuring consistent colorimetric classification.

## 4. Conclusions

In conclusion, we present a new chameleon-inspired colorimetric (CIC) sensor that seamlessly integrates a PEDOT:PSS vapor sensor with a thermochromic liquid crystal (TLC) display. The sensor’s design flexibility, electrical characteristics, and diverse sensing capabilities were comprehensively explored. The CIC sensor demonstrates remarkable proficiency in measuring ambient humidity across a wide range (20% to 90% RH) with distinct resistance sensitivities of 9.7 mΩ/%RH, 0.5 Ω/%RH, and 5.7 Ω/%RH at different humidity levels. Additionally, the sensor exhibits a linear temperature change with a sensitivity of −0.04 °C/%RH and a colorimetric temperature sensitivity of −82,036 K/%RH at lower humidity levels and −514 K/%RH at higher humidity levels. Real-time measurements of ethanol vapor with varying concentrations further showcase the CIC sensor’s applicability in gas sensing applications. The sensor’s portability and flexibility are highlighted, making it a promising tool for real-time environmental monitoring. The integration of the PEDOT:PSS/25 wt% EG composite into the CIC sensor significantly enhances detection sensitivity to humidity, offering a cost-effective and user-friendly alternative to traditional methods. Unlike most studies that rely on expensive external instruments, the CIC sensor leverages a smartphone or camera for image capture and analysis, providing practical detection sensitivity to humidity and real-time detection capabilities for ethanol vapors. In addition, the CIC sensor presented in this work not only expands the landscape of humidity sensing research but also holds promise for diverse applications, particularly in the fields of environmental monitoring and gas sensing.

## Figures and Tables

**Table 1:** Comparison of performances of CIC sensor in relation to previously reported humidity sensors.

Sensing Material	Sensing Technique	Relative Humidity Range	Maximum Sensitivity	Measurement Instrument	Reference
GO	Capacitance/impedance	25–85%	1.22 nF/%RH	LCR meter	[20]
ZnO	QCM	5–97%	97 Hz/%RH	QCM	[39]
PEDOT:PSS/PVA	QCM	40–60%	35 Hz/%RH	QCM	[17]
ZnO	Lamb wave	10–85%	20 kHz/%RH	SAW analyzer	[41]
Ti_3_C_2_/TiO_2_	Capacitance	7–97%	1614 pF/%RH (>33% RH)	Impedance analyzer	[40]
PEDOT:PSS	Piezo resistance	20–66%	4.3 mΩ/%RH	Digital multimeter	[18]
PEDOT:PSS/PVA	Impedance/capacitance	0–80%	81 Ω/%RH 33 pF/%RH	LCR meter	[16]
**PEDOT:PSS/EG**	**Resistance/colorimetry**	**20–90%**	**0.5 Ω/%RH (RH 30 to 80%)** **5.7 Ω/%RH (RH 80 to 90%)** **−0.04 °C/%RH (all ranges)** **−82,036 K/%RH (RH 20 to 30%)** **−514 K/%RH (RH 30 to 90%)**	**Digital multimeter** **Camera** **Eye**	**This work**

## Data Availability

The data presented in this paper are available upon request from the corresponding authors.
